# Aflatoxin B1 Induces Reactive Oxygen Species-Mediated Autophagy and Extracellular Trap Formation in Macrophages

**DOI:** 10.3389/fcimb.2017.00053

**Published:** 2017-02-23

**Authors:** Yanan An, Xiaochen Shi, Xudong Tang, Yang Wang, Fengge Shen, Qiaoli Zhang, Chao Wang, Mingguo Jiang, Mingyuan Liu, Lu Yu

**Affiliations:** ^1^Key Laboratory for Zoonosis Research, Ministry of Education, Institute of Zoonosis, First Hospital of Jilin University, College of Veterinary Medicine and College of Animal Science, Jilin UniversityChangchun, China; ^2^Key Lab for New Drug Research of TCM, Research Institute of Tsinghua University in ShenzhenShenzhen, China; ^3^Guangxi Colleges and Universities Key Laboratory of Utilization of Microbial and Botanical Resources, Guangxi University for NationalitiesNanning, China; ^4^Jiangsu Co-innovation Center for Prevention and Control of Important Animal Infectious Diseases and ZoonosesYangzhou, China

**Keywords:** Aflatoxin B1, reactive oxygen species, autophagy, extracellular traps, macrophages

## Abstract

Aflatoxins are a group of highly toxic mycotoxins with high carcinogenicity that are commonly found in foods. Aflatoxin B1 (AFB1) is the most toxic member of the aflatoxin family. A recent study reported that AFB1 can induce autophagy, but whether AFB1 can induce extracellular traps (ETs) and the relationships among innate immune responses, reactive oxygen species (ROS), and autophagy and the ETs induced by AFB1 remain unknown. Here, we demonstrated that AFB1 induced a complete autophagic process in macrophages (MΦ) (THP-1 cells and RAW264.7 cells). In addition, AFB1 induced the generation of MΦ ETs (METs) in a dose-dependent manner. In particular, the formation of METs significantly reduced the AFB1 content. Further analysis using specific inhibitors showed that the inhibition of either autophagy or ROS prevented MET formation caused by AFB1, indicating that autophagy and ROS were required for AFB1-induced MET formation. The inhibition of ROS prevented autophagy, indicating that ROS generation occurred upstream of AFB1-induced autophagy. Taken together, these data suggest that AFB1 induces ROS-mediated autophagy and ETs formation and an M1 phenotype in MΦ.

## Introduction

Aflatoxins are a group of mycotoxins that are secondary metabolites of *Aspergillus flavus* and *Aspergillus parasiticus* (Kasoju et al., 2012). The commodities contaminated by aflatoxins range from daily foodstuffs to crops. Aflatoxins can enter daily life easily, especially when the humidity is high. Aflatoxins have high toxicity and carcinogenicity. The most toxic member of the aflatoxin family is aflatoxin B1 (AFB1). AFB1 contributes to human hepatocellular carcinoma (Daly et al., 2000). There are few reports examining the relationship between AFB1 and the innate immune response.

Macrophages (MΦ) are found in various tissues and play a crucial role in both the innate and adaptive immune systems. They are capable of recognizing and engulfing microbial pathogens or their toxins through phagocytosis. Lots of research papers on the effect of AFB1 on macrophages were published from 1970s (Michael et al., 1973; Richard and Thurston, 1975) until recent years (Bianco et al., 2012; Bruneau et al., 2012). And these results showed that macrophages were participate in dealing with AFB1 toxic response. Therefore, MΦ are the first line of defense against invasion (Plowden et al., 2004; Liu et al., 2014; Fejer et al., 2015). To address phagocytosed microbes, MΦ combine oxidative, and non-oxidative microbicidal mechanisms; however, these classical mechanisms are not adequate for microbes that have evolved various strategies that interfere with phagocytosis (Lloberas and Celada, 2002; Liu et al., 2014).

Autophagy is an essential intracellular process in which cytoplasmic components are delivered to the autophagosomes and lysosomes for degradation (Mihalache and Simon, 2012; Wirawan et al., 2012). Autophagy plays an essential role in the innate immune system in defense against viral and bacterial infection (Takenouchi et al., 2009) or toxins (Gutierrez et al., 2007). The classic intracellular signaling mechanism of autophagy relies on two ubiquitin-like conjugation systems involving the autophagy-related genes Atg7–Atg12–Atg5 or Atg4–Atg7–Atg8. Atg6 (Beclin-1 in mammals) plays an important role in the two systems by forming an early complex containing class III phosphoinositide 3-kinase, followed by autophagosome formation (Yuan et al., 2012). In addition, MEK/ERK is an important signaling pathway regulating autophagy via regulation of Beclin-1 (Wang et al., 2009). Reactive oxygen species (ROS) have also been suggested to positively regulate autophagy in phagocytic cells (Huang et al., 2009). A recent study reported that AFB1 can induce autophagy and ROS (Paul et al., 2015).

A phagocytosis-independent innate immune mechanism known as extracellular traps (ETs) has been recognized. Many innate effector cells use this mechanism, including neutrophils, mast cells, eosinophils and MΦ. ETs are fiber-like extracellular structures involved in the response to infections or toxins (Liu et al., 2014). ETs underlie a novel type of cell death also named ETosis. Extracellular DNA (eDNA), elastase, histone, and myeloperoxidase (MPO) are the components of ETs (Chow et al., 2010; Liu et al., 2014). NADPH oxidase (Nox2)-dependent (such as phorbol 12-myristate 13-acetate (PMA)-induced ETs) or NOX2-independent (such as ionomycin-induced ETs) oxidative bursts have been reported to activate ETosis (Remijsen et al., 2011). ETs contribute to the capture of bacteria, fungi or their toxins and provide a site for the accumulation of antimicrobial molecules to kill microbes and degrade toxins (Brinkmann et al., 2004; Fuchs et al., 2007).

Macrophage activation has a variety of phenotypes. Macrophage polarization is a classic phenomenon commonly referred to as classically (M1) and alternatively (M2) activated macrophages (Liu et al., 2015). M1 macrophages can produce pro-inflammatory mediators such as interleukin (IL)-β, tumor necrosis factor (TNF)-α, and IL-6 to mediate antimicrobial and antitumour immunity. However, M2 macrophage release anti-inflammatory molecules such as IL-10, transforming growth factor (TGF)-β1 and IL-4 and play a role in parasite containment and wound healing (Cao et al., 2015). The expression levels of CD80, CD86, iNOS, and CCR7 are much higher in M1 macrophages, whereas the expression levels of CD163, Arg-1, and CD206 are much higher in M2 macrophages (Cao et al., 2015; Liu et al., 2015; Nandakumar et al., 2016).

Previous reports investigating AFB1 have mainly focused on its toxicity and carcinogenicity. It has also been suggested that AFB1 can induce the generation of intracellular ROS such as superoxide anion (O2^•−^), hydroxyl radical (HO^•^), and hydrogen peroxide (H_2_O_2_) in mammalian cells (Sohn et al., 2003; Towner et al., 2003). In this study, we address whether autophagy and ETs can be induced by AFB1 and the possible relationship, function and mechanism among ROS, autophagy and ETs under AFB1 treatment in MΦ.

## Materials and methods

### Antibodies, chemicals, plasmids, and AFB1

The primary antibodies used for western blotting and immunofluorescence (anti-elastase antibody, anti-histone H3 antibody, anti-myeloperoxidase antibody, anti-CCR7 antibody, anti-CD163 antibody, anti-CD86 antibody, anti-CD206 antibody), anti-iNOS antibody, anti-Arg-1 antibody, and the antibodies of flow cytometry (mouse anti-human CCR7-FITC, mouse anti-human CD86-FITC, mouse anti-human CD163-PE, and mouse anti-human CD206-PE) in this study were all purchased from Cell Signaling Technology (Massachusetts, USA). Both horseradish peroxidase-labeled goat anti-rabbit and anti-mouse secondary antibodies were obtained from Beyotime (Jiangsu, China). The immunofluorescence secondary antibodies were obtained from Biolegend (San Diego, USA). Other chemicals were purchased from Dingguo Changsheng (Beijing, China). The plasmids used in our study were from *Escherichia coli*. AFB1 (purity > 98%) was purchased from Sigma (Missouri, USA).

### Cells, cell culture, and transfection

THP-1 cells and RAW264.7 cells were obtained from ATCC (Maryland, USA). THP-1 cells were maintained in RPMI 1640 medium, and RAW264.7 cells were maintained in DMEM. Both media were supplemented with 10% fetal bovine serum (FBS). All cell culture reagents were purchased from Gibco Laboratories (Gibco, NY, USA). The cells were incubated at 37°C and 5% CO_2_. Differentiation of THP-1 cells into MΦ-like cells was induced with 16 nM PMA (Sigma, Missouri, USA). For western blotting, cells were incubated in 6-well flat-bottom plates (1 × 10^6^ cells/well). For fluorescence microscopy, cells were incubated in 24-well glass-bottom plates (2 × 10^5^ cells/well). For some experiments, RAW264.7 cells were transiently transfected with GFP-LC3, RFP-LC3, or RFP-GFP-LC3 plasmid.

### Treatment with AFB1

AFB1 was diluted with cell culture medium to concentrations from 0.03 to 2 μM. Cells were cultured in serum-free and antibiotic-free RPMI 1640 or DMEM medium for 12 h before treatment with different concentrations of AFB1 for 1.5 h. AFB1 at a concentration at 0.25 μM was added to cells cultured under identical conditions for 0, 10, 30, 60, 90, 180, 360, or 720 min.

### Cell viability assay

After counting, cells at a density of 2 × 10^4^/ml were cultured in a 96-well cell culture plate (Costar) with 200 μl of RPMI 1640 per well. The cells were stimulated with 0.25 μM AFB1 at 37°C for 2 h in the presence of 5% CO_2_, followed by the addition of 10 μL/well CCK-8 (Cell Counting Kit-8, Dojindo Laboratories, Kumamoto, Japan) solutions. The plates were then incubated for 2 h in the dark. The cells without AFB1 in medium alone served as positive controls. Then, the absorbance of the samples at 450 nm was measured.

### Fluorescence microscopy

Both LC3 puncta and MΦ extracellular trap (MET) generation were visualized by fluorescence microscopy. For the former, cells were grown in 24-well glass-bottom cell-culture dishes (Nest, Jiangsu, China). Following transfection with GFP-LC3, GFP-LC3, or RFP-GFP-LC3 plasmid for 12 h, the cells were treated with AFB1 at different concentrations for 2 h, and then LC3 puncta were observed under an Olympus BX53 fluorescence microscope (Olympus, Tokyo, Japan) with a 20 × objective lens. For the MET generation study, cells were grown in 24-well glass-bottom cell culture dishes in serum-free and antibiotic-free medium for 12 h. The cells were then exposed to different concentrations of AFB1 for 2 h. The cells were stained with 5 μM SYTOX Orange (Sigma, Missouri, USA) for 10 min and then stained with 1 μM Hoechst 33342 (Sigma, Missouri, USA) for 5 min. To study the components of the METs, after incubation, the slides were fixed with 4% paraformaldehyde solution for 30 min at 4°C. Blocking was performed with 5% BSA for 1 h at room temperature (RT). After washing three times, the slides were incubated with primary antibody at 4°C overnight. After washing three times, the slides were incubated with the secondary antibody for 1 h at RT. To label the DNA, the cells were incubated with Hoechst 33342 (1 μM) for 5 min. Images were collected using fluorescence microscopy.

### Western blotting

Cells treated with AFB1 were harvested in cold PBS and centrifuged at 3,000 g for 5 min at 4°C. The collected cells were incubated on ice with RIPA lysis buffer (Sigma, Missouri, USA) containing 1 mM PMSF (Sigma, Missouri, USA) for 10 min. The supernatant of the lysates was obtained by centrifugation at 12,000 g for 10 min at 4°C. A BCA protein assay kit (Beyotime, Jiangsu, China) was used to quantify the concentration of protein. Equal amounts of protein were separated on a 15% polyacrylamide gel and transferred onto polyvinylidene fluoride (PVDF) membranes (Beyotime, Jiangsu, China). The membranes were blocked for 2 h in blocking buffer (5% non-fat milk, 0.1% Tween 20 and TBS) and incubated with primary antibodies at 4°C overnight, followed by the peroxidase-conjugated secondary antibody for 2 h. The corresponding bands were detected using an enhanced chemiluminescence detection kit (Beyotime, Jiangsu, China). The images were collected by a CanoScan LiDE 100 scanner (Canon, Tokyo, Japan). Protein blots were measured with software ImageJ.

### Detection of cytosolic ROS

Dihydrorhodamine (DHR) 123 (Sigma, Missouri, USA), a fluorescent indicator of cytosolic ROS, was used to detect cytosolic ROS. Cells were preloaded with 1 μM DHR 123 for 20 min without light. Then, the cells were pretreated with DPI and stimulated with the indicated reagents. The results were obtained using a plate reader.

### Flow cytometry

Cell staining and flow cytometer analysis were performed as described (Mandal et al., 2011). Briefly, differentiated M1 and M2 macrophages were characterized by staining with the following antibodies: mouse anti-human CCR7-FITC, mouse anti-human CD86-FITC, mouse anti-human CD163-PE, and mouse anti-human CD206-PE. The samples were detected with a FACSCalibur flow cytometer (BD Biosciences, San Jose, CA). The data acquired were analyzed with FlowJo (Treestar Software, Ashland, OR, USA).

### Degradation of AFB1

AFB1 degradation was conducted as previously described with some modifications (Brinkmann et al., 2004). Briefly, cells were seeded in 24-well flat-bottom plates and treated with cytochalasin D for 20 min. Culture medium without cells was added to the control well. The DPI-pretreated cells were then exposed to the indicated reagents for 8 h. For autophagy, cells, and Atg7-silenced cells were seeded in 24-well flat bottom plates and treated with AFB1 for 8 h. AFB1 degradation was detected using the RIDASCREEN AFB1 30/15 enzyme-linked immunosorbent assay (ELISA; R Biopharm) in accordance with the manufacturer's instructions.

### Statistical analysis

All results were expressed as the mean ± SD. Group means were compared using one-way ANOVA with Bonferroni adjustment using GraphPad Prism version 5.0b software to determine statistical differences. *P*-values of 0.05 or less were considered statistically significant.

## Results

### AFB1 induced a time- and dose-dependent autophagic response

**Figure 1:**
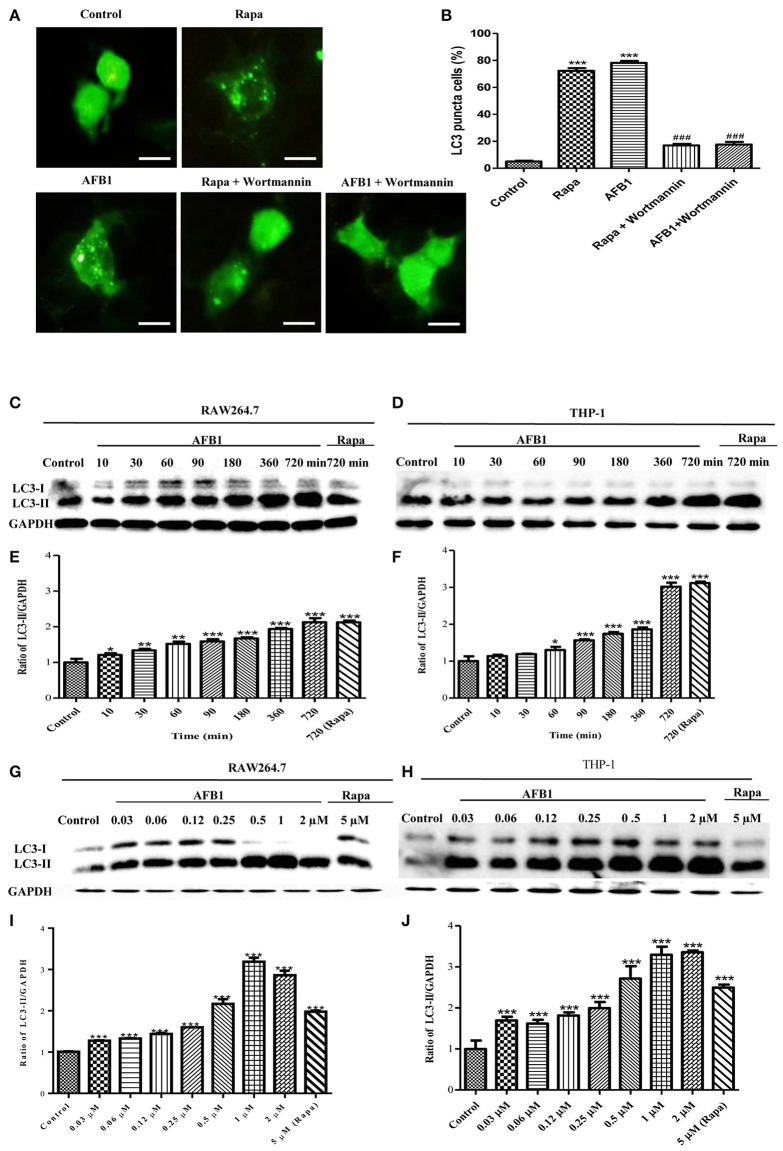
**AFB1 induced a time- and dose-dependent autophagic response. (A)** RAW264.7 cells were transfected with GFP-LC3 plasmid for 12 h. The cells were pretreated with Rapa (5 μM, 12 h) and wortmannin (100 nM, 1 h) and then treated with AFB1 (0.25 μM) for 2 h. Scale bars = 20 μm. **(B)** The percentage of GFP-LC3 puncta cells was calculated. ^***^P < 0.001 compared with the control groups; ^###^*P* < 0.001 compared with Rapa and AFB1. **(C,D)** RAW264.7 cells and THP-1 cells were pretreated with 5 μM Rapa for 12 h and then treated with 0.25 μM AFB1 for different times. **(G,H)** The two cell lines were similarly treated with Rapa and subsequently exposed to different concentrations of AFB1 for 1.5 h. **(E,F**, **I,J)** Western blotting was conducted to assay the level of LC3. The ratio of LC3-II/GAPDH was calculated. ^*^P < 0.05, ^**^P < 0.01, ^***^P < 0.001 compared with the control groups in the same cell line. The data are representative of three experiments with similar results.

To investigate whether AFB1 induced autophagy, we transfected the GFP-LC3 plasmid into RAW264.7 cells. Following successful transfection, RAW264.7 cells were treated with AFB1 for 2 h. We also treated the transfected cells with the autophagy activator rapamycin (Rapa, 5 μM) or the inhibitor wortmannin (100 nM). The results obtained by fluorescence microscopy showed that there was a significant increase in LC3 puncta in most AFB1-treated RAW264.7 cells. Rapa also induced an increase in LC3 puncta, whereas wortmannin pretreatment significantly decreased the LC3 puncta in rapamycin- and AFB1-treated cells (Figures 1A,B). This result suggested that AFB1 might induce an autophagic response.

To further confirm the autophagy induced by AFB1, western blotting was used to assay the LC3 levels of AFB1-treated MΦ (THP-1 and RAW264.7). THP-1 and RAW264.7 cells were incubated in 6-well flat-bottom plates and treated with AFB1 or Rapa at concentrations of 0.03–2 μM for 7 time points (10–720 min). The results showed that the ratio of LC3-II/GAPDH was significantly increased in AFB1-treated THP-1 and RAW264.7 cells compared with untreated cells in a time-dependent (Figures 1C–F) and dose-dependent manner, and the expression of LC3 reached its peak when cells were treated with AFB1 for 1 μM (Figures 1G–J). These data confirmed that AFB1 could induce autophagy in MΦ cells.

### AFB1 induced a complete autophagic process

**Figure 2:**
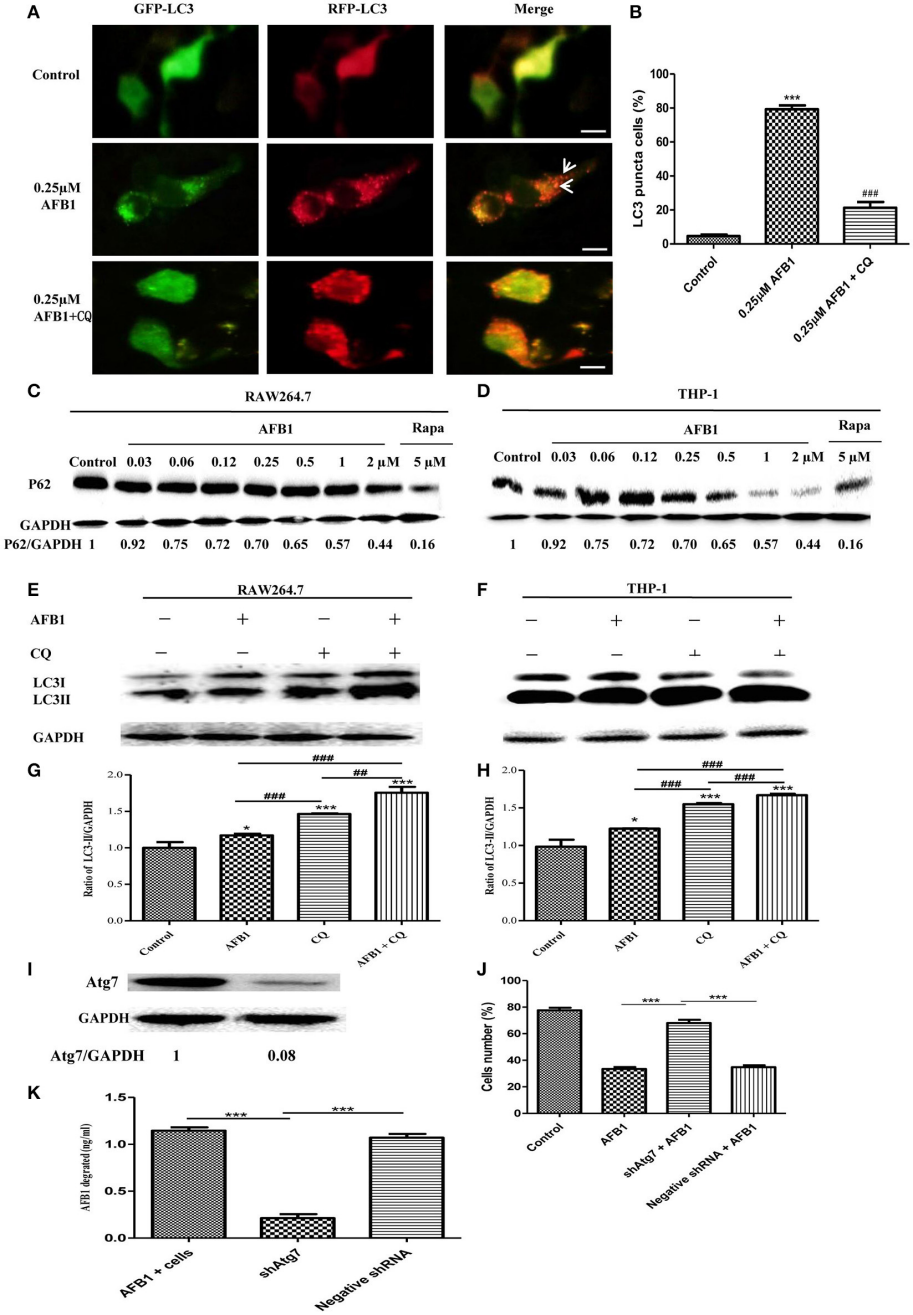
**AFB1 induced a complete autophagic process. (A)** RAW264.7 cells were transfected with tandem GFP-RFP-LC3 plasmid for 12 h. Cells were pretreated with or without CQ, followed by treatment with AFB1 (0.25 μM) for 2 h and observation by fluorescence microscopy. Scale bars = 20 μm. **(B)** The percentage of GFP-RFP-LC3 puncta cells was calculated. ^***^P < 0.001 compared with the control groups; ^###^*P* < 0.001 compared with AFB1. **(C,D)** RAW264.7 cells and THP-1 cells were incubated in 6-well flat-bottom plates (1 × 10^6^ cells/well) and cultured in serum-free and antibiotic-free medium for 12 h. Following treatment with Rapa (5 μM, 12 h), the cells were treated with different concentrations of AFB1 from 0.03 to 2 μM for 1.5 h. Western blotting of SQSTM1 was performed. **(E,F)** RAW264.7 cells and THP-1 cells were pretreated with CQ (20 μM) for 1 h and subsequently exposed to AFB1 (0.25 μM) for 1.5 h. Western blotting for LC3 was then performed. **(G,H)** The ratios of p62/GAPDH and LC3-II/GAPDH were calculated. ^*^P < 0.05, ^***^P < 0.001 compared with the control groups of the same cell line; ^##^*P* < 0.01, ^###^*P* < 0.001. **(I)** Western blotting of Atg7 in RAW264.7 cells with or without knockdown treatment. **(J)** Cell viability was estimated using the Cell Counting Kit-8 (CCK-8) assay ^***^P < 0.001. **(K)** Non-transfected RAW264.7 cells, Atg7-silenced cells, and cells transfected with the shRNA negative control cells were incubated in 24-well plates (2 × 10^5^ cells/well) with serum-free medium for 12 h, followed by treatment with AFB1 (2 μM) for 8 h. We performed AFB1 ELISA to determine the AFB1 content using an ELISA kit. ^***^P < 0.001. The data are representative of three experiments with similar results.

Although AFB1 induced autophagy, whether it was a complete process was unclear. To examine this possibility, RAW264.7 cells were transfected with RFP-GFP-LC3 plasmid and treated with AFB1. The GFP moiety of this tandem autophagosome marker is sensitive to lysosomal proteolysis and quenching in acidic pH, whereas RFP is not. Therefore, the green fluorescent component of the composite yellow fluorescence of this RFP-GFP-LC3 reporter is lost after autophagosome fusion with lysosomes (Sun et al., 2012). Our results showed that for the AFB1-treated groups and the groups pretreated with chloroquine (CQ), the AFB1-treated groups contained more green, and red LC3 puncta than the control groups. Most importantly, the number of red puncta was significantly higher than the number of green puncta (Figures 2A,B), indicating that some green puncta had vanished during the autophagic process and suggesting that AFB1 induces a complete process.

We also measured the degradation of the polyubiquitin-binding protein p62/sequestosome 1 (SQSTM1), a marker for the autophagy-mediated protein degradation pathway (Pei et al., 2014). Using different concentrations of AFB1 to assess THP-1 cells and RAW264.7 cells, we found that the levels of the SQSTM1 protein decreased with increasing AFB1 concentration (Figures 2C,D). This result is consistent with a previous report that the degradation of SQSTM1 indicated a complete autophagic response (Pei et al., 2014). In addition, we treated THP-1 cells and RAW264.7 cells with CQ, a lysosomotropic agent that inhibits autophagosome fusion with lysosomes and therefore suppresses autophagic degradation. Our results showed that LC3-II accumulated upon CQ treatment (Figures 2E–H), indicating that autophagosome flux and the degradation of LC3-II by lysosomal proteolysis was inhibited. Moreover, the level of LC3-II was obviously higher after treatment with the combination of AFB1 and CQ compared with AFB1 treatment alone (Figures 2E–H), demonstrating that autophagy induced by AFB1 included the process of the fusion of autophagosomes with lysosomes. Taken together, these data suggested that AFB1 induced a complete autophagic process. To further corroborate whether AFB1-induced autophagy is a cell death-mediating mechanism, we first used a specific shAtg7 plasmid to knockdown the expression of Atg7 (Autophagy related genes) (Figure 2I). The cell viability of AFB1-treated RAW264.7 cells transfected with the shAtg7 plasmid was significantly increased compared with cells transfected with the negative shRNA plasmid and non-transfected cells (*P* < 0.05) (Figure 2J). This result confirmed that the AFB1-induced autophagic response was a cell death-mediating mechanism. To study the role of autophagy, the AFB1 content was detected by ELISA. ELISA revealed that the content of AFB1 (the original treatment concentration of AFB1 was 2 μM) significantly decreased after 8 h incubation with non-transfected or negative shRNA cells compared with Atg7-silenced cells under AFB1 treatment (*P* < 0.001) (Figure 2K).

### The autophagy response induced by AFB1 was MEK/ERK-dependent and upregulated beclin-1

**Figure 3:**
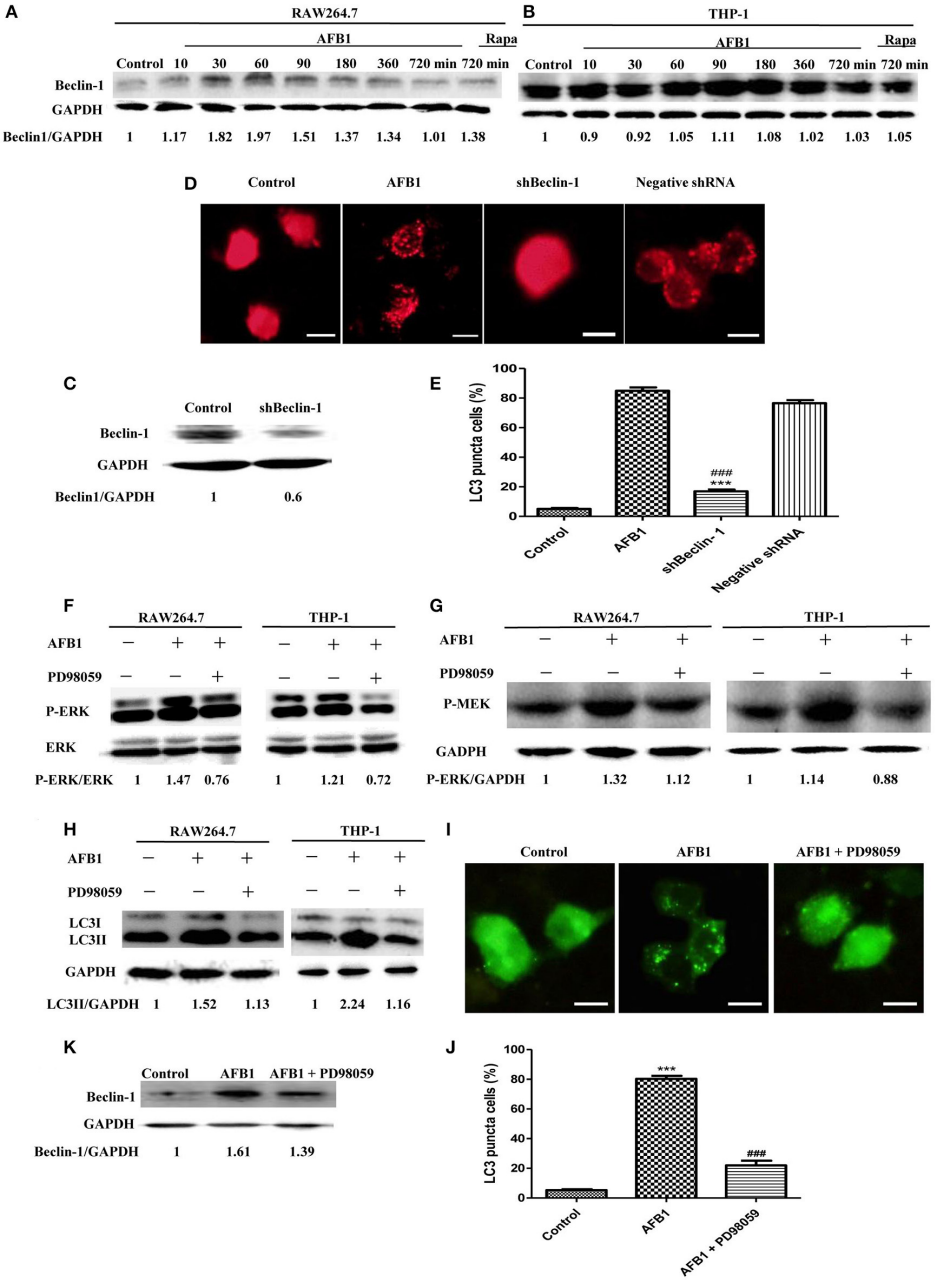
**The autophagy response induced by AFB1 was MEK/ERK-dependent and upregulated Beclin-1. (A,B)** RAW264.7 cells and THP-1 cells were pretreated with Rapa (5 μM) for 12 h and then treated with AFB1 for different times. Western blotting was used for the Beclin-1 protein assay. The ratios of Beclin-1/GAPDH were calculated. **(C)** Western blotting of Beclin-1 in RAW264.7 cells with or without knockdown treatment was conducted. **(D)** RAW264.7 cells were transfected with the RFP-LC3 plasmid for 12 h and then transfected with the shRNA negative control or shBeclin-1 plasmids for 48 h before the cells were infected with AFB1 (0.25 μM) for 2 h. Fluorescence images show the induction of LC3 puncta. Scale bars = 20 μm. **(E)** The percentage of RFP-LC3 puncta cells was calculated. ^***^P < 0.001 compared with AFB1-infected cells; ^###^*P* < 0.001 compared with negative control shRNA plasmid. **(F–H)** RAW264.7 cells and THP-1 cells were pre-treated with the MEK/ERK inhibitor PD98059 (20 μM) for 1 h and then treated with AFB1 (0.25 μM) for P-ERK, P-MEK, and LC3 protein assays. **(I)** RAW264.7 cells were transfected with GFP-LC3 plasmid for 12 h. The cells were pretreated with PD98059 (20 μM, 1 h) and then treated with AFB1 (0.25 μM) for 2 h. Scale bars = 20 μm. **(J)** The percentage of GFP-LC3 puncta cells was calculated. ^***^P < 0.001 compared with the control groups; ^###^*P* < 0.001 compared with AFB1. **(K)** Western blotting of Beclin-1 was performed after treatment with PD98059 (20 μM, 1 h) and AFB1 (0.25 μM).

To analyze the possible mechanism of the autophagic response induced by AFB1, we detected the expression level of Beclin-1 in AFB1-treated THP-1 and RAW264.7 cells. AFB1 treatment increased the expression of Beclin-1 in the two cell lines, and the expression of Beclin-1 was maximal when the cells were treated with AFB1 for 1 h (Figures 3A,B). This result suggested that the AFB1-induced autophagic response might be mediated by Beclin-1. Next, we used a specific shBeclin-1 plasmid to knockdown the expression of Beclin-1 (Figure 3C) to further corroborate the correlation of Beclin-1 and AFB1-induced autophagy. RPF-LC3 puncta were significantly decreased in AFB1-treated RAW264.7 cells transfected with the shBeclin-1 plasmid compared with the group transfected with the negative shRNA plasmid and the control groups (*P* < 0.05) (Figures 3D,E). This result verified that the AFB1-induced autophagic response was mediated by Beclin-1.

Additionally, our western blotting analysis showed that AFB1 treatment significantly increased the phosphorylation of MAP kinase kinase 1/2 (MEK1/2) and extracellular signal-regulated kinases 1/2 (ERK1/2), and treatment with AFB1 in combination with the MEK inhibitor PD98059 markedly abolished the expression of MEK1/2 and ERK1/2 in THP-1 and RAW264.7 cells (Figures 3F,G). To further verify whether the activation of MEK/ERK was related to AFB1-induced autophagy, THP-1, and RAW264.7 cells were also treated with AFB1 together with the MEK inhibitor PD98059. Western blotting results for LC3 showed that the inhibition of MEK/ERK by PD98059 abrogated the autophagic response (Figure 3H). Moreover, the fluorescence microscopy results also showed that inhibiting MEK/ERK by PD98059 suppressed the punctate GFP-LC3 pattern induced by AFB1 treatment (Figures 3I,J). These data indicated that the autophagy response caused by AFB1 is MEK/ERK-dependent.

Furthermore, to explore the order of precedence of Beclin-1 and MEK/ERK in the autophagy response induced by AFB1, we assayed the expression of Beclin-1 in RAW264.7 cells treated with AFB1 in combination with the MEK inhibitor PD98059. The inhibition of MEK/ERK activation abrogated the up-regulation of Beclin-1 caused by AFB1 treatment (Figure 3K). This result suggested that the activation of MEK/ERK in response to autophagy stimuli induced by AFB1 treatment upregulated Beclin-1.

### AFB1 induced the generation of METs

**Figure 4:**
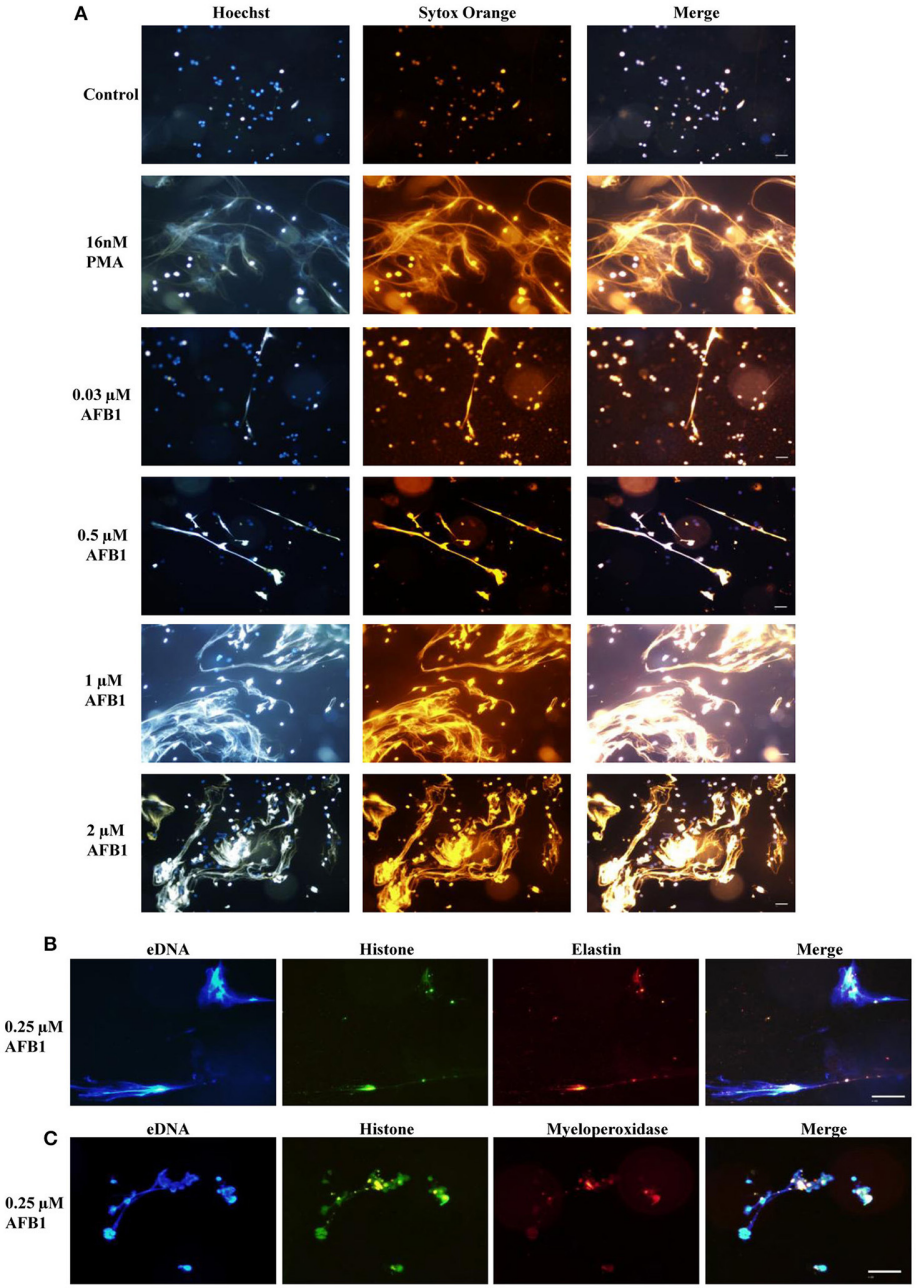
**AFB1 induced the generation of METs. (A)** THP-1 cells were incubated in 24-well glass-bottom plates (2 × 10^5^ cells/well) with serum-free RPMI 1640 for 12 h. Cells were treated with PMA (16 nM) and AFB1 (2 μM) at concentrations of 0.03, 0.12, 0.5, or 2 μM for 2 h. METs were stained with SYTOX Orange (5 mM) for 10 min, and nuclei were stained with Hoechst 33342 (1 μM) for 5 min. **(B)** Histone, elastin, and eDNA colocalized. **(C)** Histone, myeloperoxidase, and eDNA colocalized. The images were obtained by fluorescence microscopy with a 20 × objective lens. Scale bars = 50 μm.

To explore whether AFB1 induced the generation of ETs in MΦ, THP-1 cells were incubated with different concentrations of AFB1 and then stained with Hoechst 33342 (1 μM) and SYTOX Orange (5 μM), followed by image collection. The results showed that AFB1 treatment induced highly reticulated structures containing extracellular DNA in a concentration-dependent manner (Figure 4A), similar to the results of PMA treatment. However, few METs formed when MΦ were incubated without AFB1 (Figure 4A). This result suggested that AFB1, as an exogenous toxin, was recognized through ETs by phagocytes. In addition, we found that elastase (anti-elastase antibody) (Figure 4B) and MPO (anti-myeloperoxidase antibody) (Figure 4C) colocalized with histone (anti-histone H3 antibody) and eDNA, respectively. These results demonstrated that elastase, MPO, histone, and eDNA were components of the AFB1-induced METs.

### Autophagy and ROS are required for AFB1-induced MET formation

**Figure 5:**
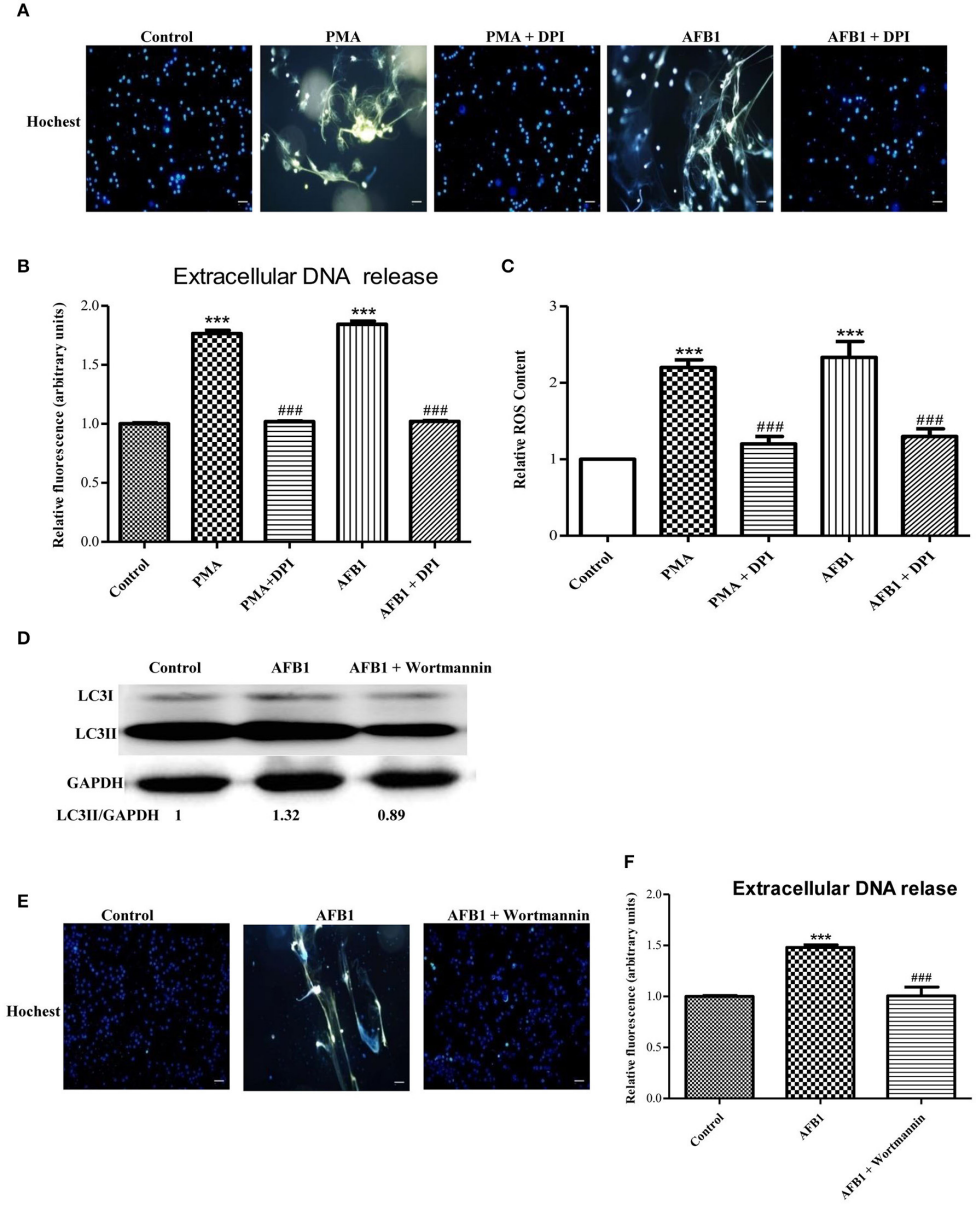
**Autophagy and ROS are required for AFB1-induced MET formation. (A)** THP-1 cells were pre-treated with the NOX2 inhibitor DPI (50 μM) for 1 h then, PMA (16 nM) and AFB1 (0.25 μM) were added to the cells for 2 h. The cells were stained with Hoechst 33342 (1 μM) and then observed by fluorescence microscopy with a 20 × objective lens. Scale bars = 50 μm. **(B)** The fluorescence intensity of extracellular DNA was detected by a plate reader. **(C)** For the quantification of cytosolic ROS, THP-1 cells were incubated in 24-well plates (2 × 10^5^ cells/well), pre-treated with DPI (50 μM) for 1 h and then treated with PMA (16 nM) and AFB1 (0.25 μM). Cytosolic ROS were labeled by DHR 123 (1 μM) and detected by a plate reader. The data were analyzed by Graphpad prism software (GraphPad, San Diego). ^***^P < 0.001 compared with the control groups in the same cell line; ^###^*P* < 0.001 compared with the PMA and AFB1. The data are representative of three experiments with similar results. **(D)** THP-1 cells were cultured in serum-free and antibiotic-free medium for 12 h. Then, cells were pretreated with wortmannin (100 nM) for 1 h followed by AFB1 (0.25 μM) for 1.5 h. Western blotting of LC3 was performed. **(E)** THP-1 cells were pre-treated with the autophagy inhibitor wortmannin (100 nM) for 1 h, and then AFB1 (0.25 μM) was added to the cells for 2 h. The cells were stained with Hoechst 33342 (1 μM) and then observed by fluorescence microscopy with a 20 × objective lens. Scale bars = 50 μm. **(F)** The fluorescence intensity of extracellular DNA was detected by a plate reader ^***^P < 0.001 compared with the control groups; ^###^*P* < 0.001 compared with the AFB1.

We also investigated the possible mechanisms involved in MET formation induced by AFB1. First, we assessed the relationship between ROS and MET formation. The NOX2 inhibitor DPI (50 μM) inhibited MET formation induced by AFB1 or PMA (Figures 5A,B). This result demonstrated that AFB1-induced METs were NOX2-dependent, similar to PMA. Next, we used a fluorescent indicator of cytosolic ROS (dihydrorhodamine or DHR 123) (Douda et al., 2015) with a plate reader assay to detect cytosolic ROS production in AFB1-treated THP-1 cells. AFB1 or PMA treatment triggered a large increase in cytosolic ROS production (Figure 5C), whereas DPI significantly inhibited the enhancement of AFB1- or PMA-induced cytosolic ROS production (Figure 5C). These results demonstrated that the AFB1-induced MET formation was dependent on the ROS produced by NOX2. To further examine the role of AFB1-induced autophagy in MET formation, we used the PI3K inhibitor wortmannin to inhibit autophagy. Pretreatment with wortmannin abolished LC3B-I to LC3B-II conversion, as expected (Figure 5D), and inhibited MET formation (Figures 5E,F). Thus, either inhibition of autophagy or ROS prevented MET formation by AFB1, indicating that autophagy and ROS are required for AFB1-induced MET formation.

### ROS generation is required for the activation of AFB1-induced autophagy

**Figure 6:**
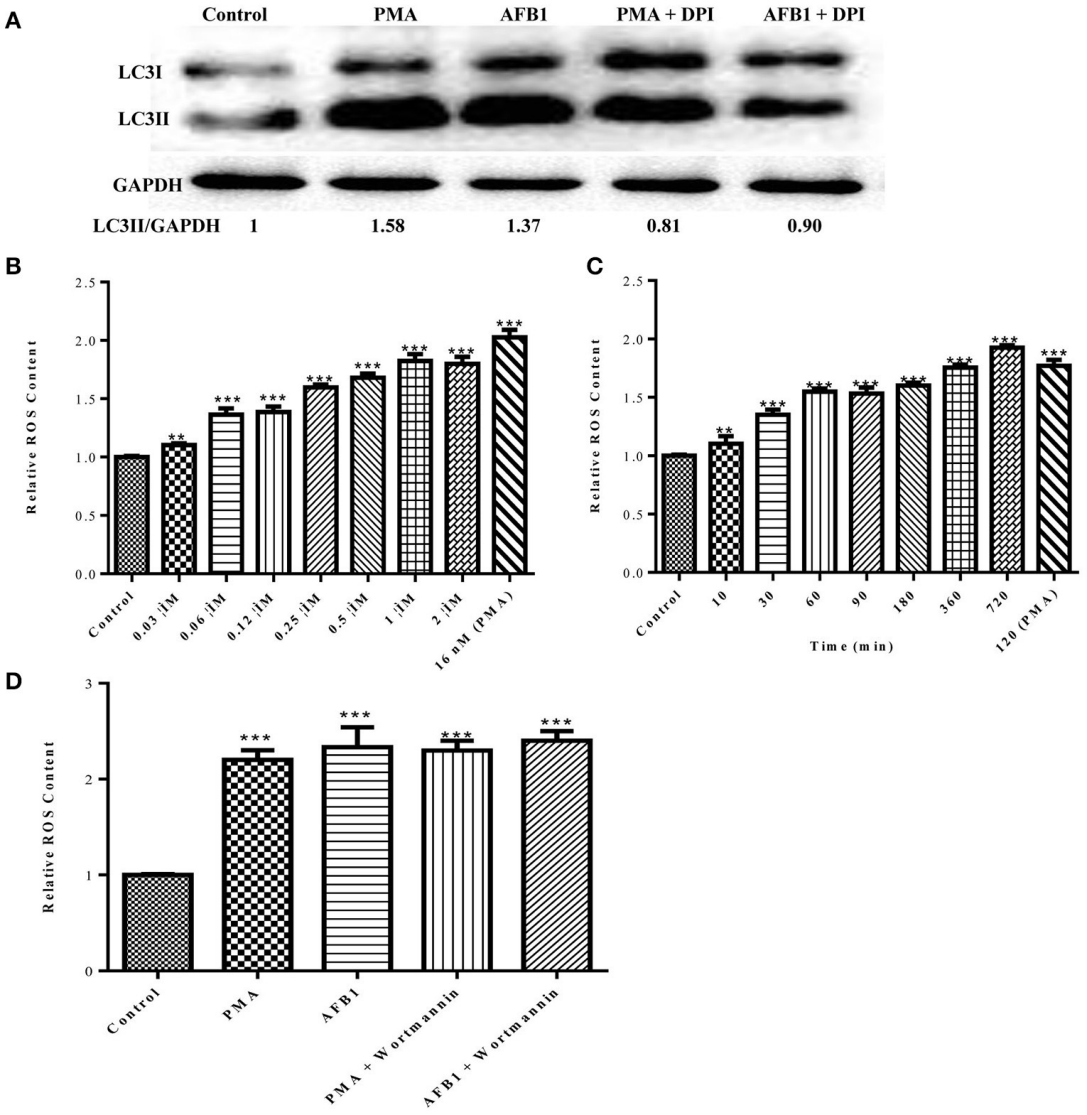
**ROS generation is required for the activation of AFB1-induced autophagy. (A)** RAW264.7 cells were pre-treated with the NOX2 inhibitor DPI (50 μM) for 1 h. PMA (16 nM) and AFB1 (0.25 μM) were then added to the cells for 2 h and western blotting of LC3 was performed. **(B,C)** RAW264.7 cells were treated with AFB1 at different concentrations and times. Cytosolic ROS were labeled by DHR 123 (1 μM) and were detected by a plate reader, ^**^P < 0.01, ^***^P < 0.001 compared with the control groups in the same cell line. **(D)** Cells were pretreated with wortmannin (100 nM, 1 h) after treatment with AFB1 (0.25 μM) and PMA (16 nM) for 2 h. Cytosolic ROS were detected by a plate reader, ^***^P < 0.001 compared with the control groups in the same cell line.

Because both autophagy and ROS are required for AFB1-induced MET formation, we further assessed the possible relationship between ROS and autophagy induced by AFB1 in MΦ. We detected the levels of cytosolic ROS and LC3 expression in AFB1-treated RAW264.7 cells with or without DPI. PMA, which induces ROS generation, was used as a positive control agent. DPI pretreatment significantly decreased the ratio of LC3-II/GAPDH under AFB1 treatment (*P* < 0.05), similar to the results for PMA (Figure 6A), whereas AFB1 induced cytosolic ROS production in a dose- and time-dependent manner (Figures 6B,C). Pretreatment with 100 nM wortmannin did not affect PMA- or AFB1-induced cytosolic ROS production (Figure 6D). Thus, inhibition of ROS prevented autophagy, indicating that ROS generation occurs upstream of AFB1-induced autophagy.

### MET formation reduced the AFB1 content

**Figure 7:**
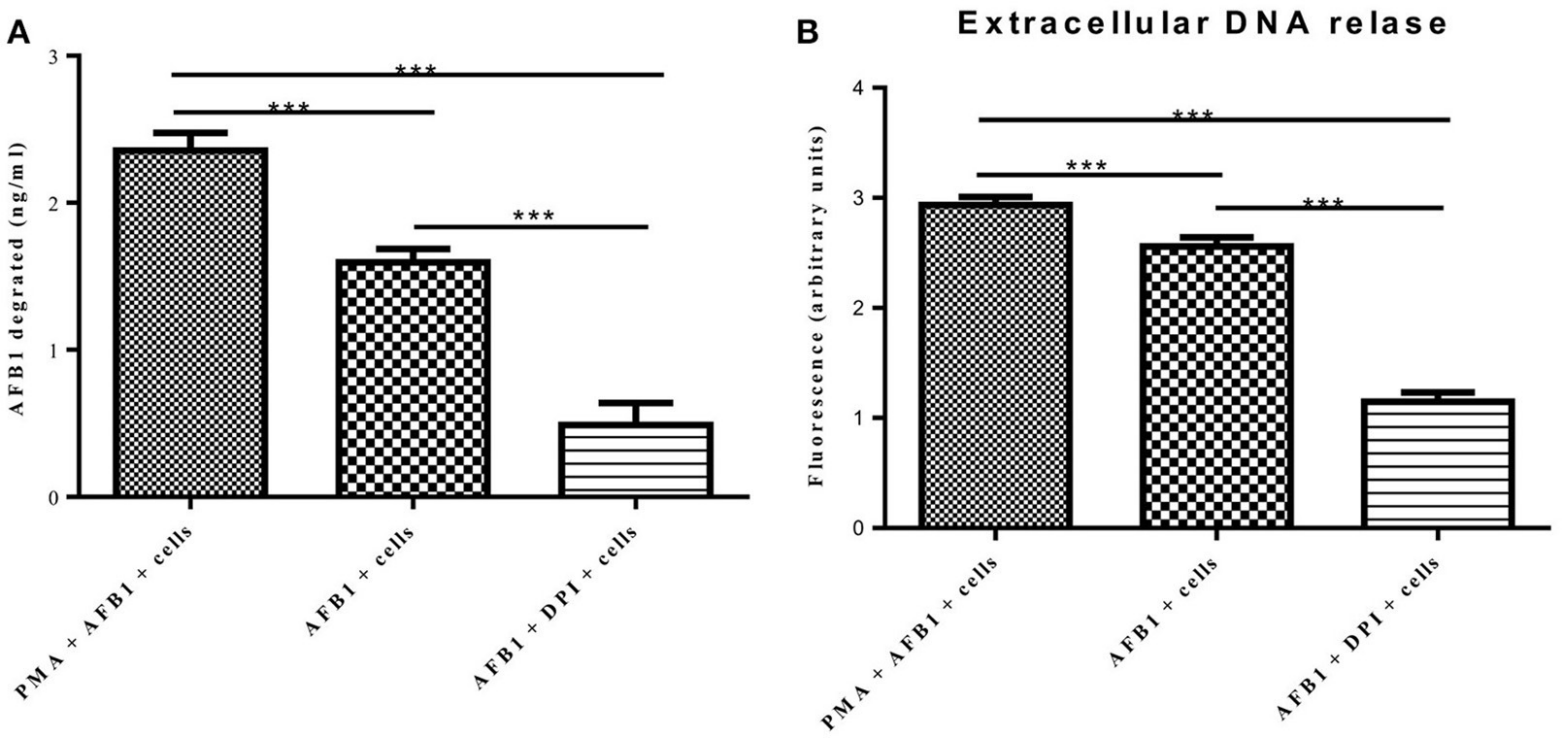
**METs formation reduced the AFB1 content. (A)** RAW264.7 cells were incubated in 24-well plates (2 × 10^5^ cells/well) with serum-free medium for 12 h. Only one of the wells was pretreated with DPI (50 μM) for 1 h, and then CytD was added to all of wells for 20 min, followed by treatment with AFB1 (2 μM) or PMA (16 nM) and AFB1 (2 μM) for 8 h. We used the AFB1 ELISA to determine the AFB1 content through an ELISA kit. The cells were treated with AFB1 with or without PMA, or AFB1 with or without DPI. ^*^P < 0.05, ^**^P < 0.01, ^***^P < 0.001 compared with the control groups (only cells) in the same cell line. The data are representative of three experiments with similar results. **(B)** The fluorescence intensity of Extracellular DNA was detected by a plate reader. The data are representative of three experiments with similar results.

A previous report determined that ET formation by phagocytes not only traps and kills microorganisms but also degrades toxins (Brinkmann et al., 2004). Because AFB1 is sensed by METs, we examined whether MET formation reduced the AFB1 content. ELISA showed that the content of AFB1 (the original treatment concentration of AFB1 was 2 μM) was significantly decreased after 8 h of incubation with RAW264.7 cells compared to incubation without cells, and PMA (16 nM) treatment significantly enhanced the degradation of AFB1 by METs (Figure 7A). In particular, the ET scavenger DPI (50 μM) significantly decreased the degradation of AFB1 by METs (Figure 7A). Further analysis revealed a strong positive correlation (*r* = 0.93) between the amount of METs and the amount of AFB1 degraded by METs (Figure 7B and Figure S1). We suggest that innate immune cells use ET formation to degrade the exogenous toxin AFB1 and weaken its toxicity.

### AFB1 induced M1-like polarization in RAW264.7 cells

**Figure 8:**
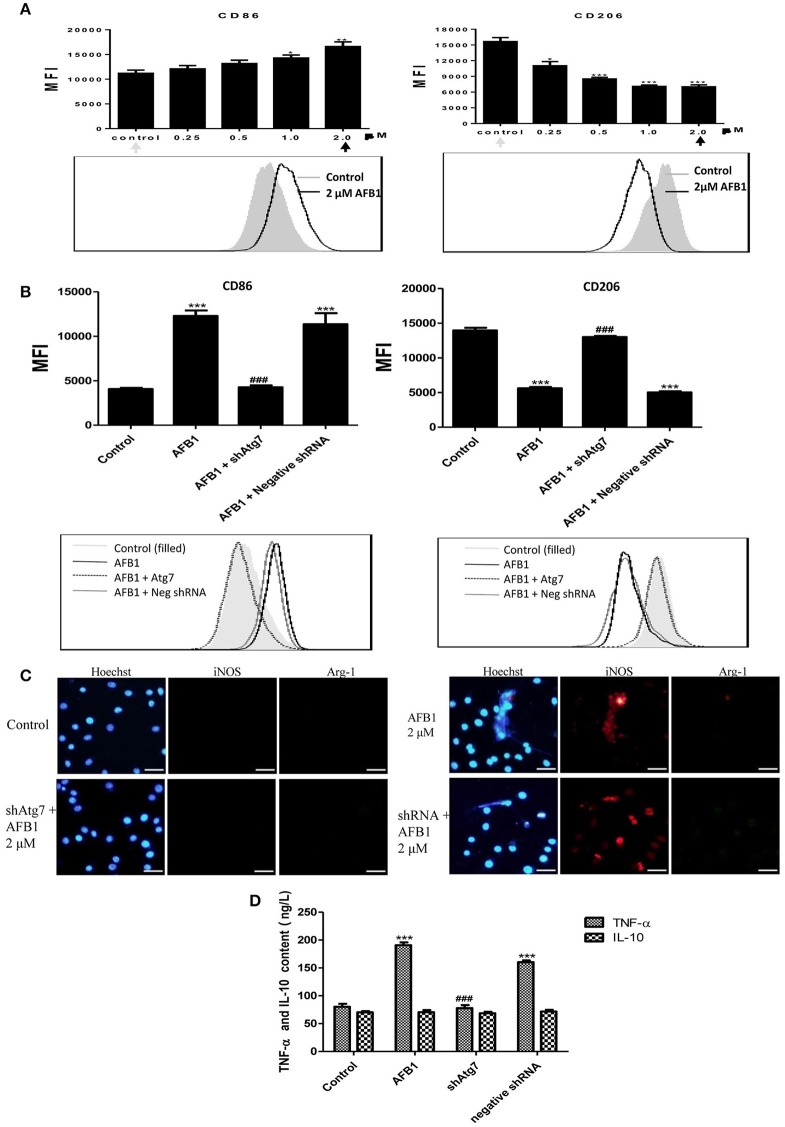
**AFB1 induced M1-like polarization in RAW264.7 cells. (A)** The expression levels of CD86 and CD206 in RAW264.7 cells were detected by flow cytometry. The results are presented as the mean fluorescence intensity (MFI). ^***^P < 0.001 compared with the control groups of RAW264.7 cells. The data are representative of three experiments with similar results. The histogram figure represents the comparison of the 2 μM AFB1-treated group and control group. **(B)** CD86 and CD206 in RAW264.7 cells, Atg7-silenced cells and cells transfected with the shRNA negative control were detected by flow cytometry. The results are presented as the mean fluorescence intensity (MFI). ^***^P < 0.001 compared with the control groups of RAW264.7 cells; ^###^*P* < 0.001 compared with AFB1-infected cells and cells transfected with the negative control shRNA plasmid. The histogram figure represents the comparison of the 2 μM AFB1-treated group, Atg7-silenced cells, cells transfected with the shRNA negative control and control group. **(C)** iNOS, Arg-1 and eDNA colocalized, and the immunofluorescence staining shows the comparison of the 2 μM AFB1-treated Non-transfected cell group, Atg7-silenced cell group, cells transfected with the shRNA negative control group and control group. **(D)** TNF-α and IL-10 secretion was measured by ELISA. ^***^P < 0.001 compared with the control group; ^###^*P* < 0.001 compared with the AFB1-infected cells and cells transfected with the negative control shRNA plasmid.

To investigate the effect of AFB1 on the polarization of macrophages, we evaluated the phenotypes of Atg7-silenced Raw 264.7 cells and non-transfected cells with or without AFB1 treatment. AFB1 treatment increased the expression of CCR7 and CD86; which is a marker of the M1 phenotype. However, CD163 and CD206 (M2 marker) were decreased by AFB1 treatment in non-transfected RAW264.7 cells (Figure 8A). Polarization to M1-like polarization occurred in a dose-dependent manner. However, Atg7-silenced cells did not exhibit a trend of M1 or M2 polarization under AFB1 treatment (Figure 8B). Additionally, we used immunofluorescence staining to examine the phenotype characteristics. CCR7 (M1 marker) was significantly more expressed than CD163 (M2 marker) (Figure S2). Furthermore, we analyzed cytokines associated with polarization in Atg7-silenced and non-transfected cells under AFB1 treatment. The immunofluorescence staining showed that iNOS expression was significantly higher than Arg-1 expression, but neither iNOS nor Arg-1 was expressed in Atg7-silenced cells (Figure 8C). In addition, ELISA showed that the expression of TNF-α was higher in non-transfected cells than in Atg7-silenced cells, and IL-10 was not significantly expressed in either non-transfected cells or Atg7-silenced cells (Figure 8D). Taken together, these results indicate that AFB1-treated RAW264.7 cells exhibited a dose-dependent increase in M1 polarity and that aflatoxin-induced autophagy affected the polarization and cytokine responses.

## Discussion

AFB1 is a coumarin derivative with mutagenic, teratogenic, and carcinogenic effects. AFB1 plays an essential role in human hepatic and extrahepatic carcinogenesis (Puiu et al., 2012). Most published studies have focused on the interaction between AFB1 and hepatocytes. However, as MΦ are first-line immune cells, we hypothesized that MΦ must respond to AFB1. Whether macrophages are needed for the AFB1 toxic response, dozens of research papers has been published to show that the effects of AFB1 on macrophages from different organizations of various animals (including those from human being) *in vitro* and *in vivo* (Schlemper et al., 1991; Mohsenzadeh et al., 2016), and effects studied of AFB1 on macrophages included the ability of macrophage to activate AFB1 (Michael et al., 1973; Richard and Thurston, 1975; Bianco et al., 2012; Bruneau et al., 2012); DNA binding, adduct characterization and metabolic activation of AFB1 catalyzed by macrophages Steinberg et al., 1990; Schlemper et al., 1991; Donnelly et al., 1996; immune functional impairment of macrophages by AFB1 containing adherence potential, morphological alterations, phagocytic activity, microbiocidal activity (Mohapatra and Roberts, 1985; Cusumano et al., 1995; Bianco et al., 2012), NO production (Moon and Pyo, 2000), mRNA and protein secretion levels of pro-inflammatory cytokines, cell surface marker expression (Dugyala and Sharma, 1996; Bruneau et al., 2012), resistance to tumor (Moon et al., 1999), and so on. All these results suggested that macrophages are needed for the AFB1 toxic response, and the work of macrophages is relevant to the disease process of AFB1 intoxication. And our results supplemented and perfected the data from other scientists in some extent.

A recent study reported that AFB1 induced autophagy and ROS (Paul et al., 2015). However, whether the autophagic response induced by AFB1 is a complete process, whether AFB1 can induce ETs, and the relationship among innate immune responses, ROS, autophagy, and ETs induced by AFB1 remain unknown. As predicted, our results showed that AFB1 treatment triggered an autophagic response in both MΦ THP-1 and RAW264.7 cells in a dose-dependent manner (0.03–2 μM) (Figures 1G–J). MET release was also induced by AFB1 in THP-1 cells in a dose-dependent manner (0.03–2 μM) (Figure 4A). In addition, we analyzed the possible mechanism of the three novel innate immune responses induced by AFB1: ROS, autophagy, and MET formation. The results reveal a new mechanism of AFB1 recognition and treatment by phagocytes *in vitro*.

Autophagy is an intracellular membrane trafficking pathway that plays an important role in controlling bacterial infection and toxins (Ham et al., 2011; Huynh et al., 2011). Bacteria or toxins in the cytoplasm are captured by autophagy and delivered to autophagosomes and autolysosomes for destruction (Yuan et al., 2012). Therefore, the final process of autophagosome fusion with lysosomes is the most important step in the treatment of pathogens and toxins. Thus, a complete process of AFB1-induced autophagy is essential for MΦ to address AFB1. SQSTM1/P62, an adaptor protein, binds LC3 and ubiquitin to facilitate autophagy of polyubiquitinated proteins and is a marker for the autophagy-mediated protein degradation pathway (Lam et al., 2012; Pei et al., 2014). Therefore, the down-regulation of SQSTM1 can be used to determine if autophagy is complete. In our study, the GFP-RFP-LC3 plasmid was used because the GFP moiety of this tandem plasmid is sensitive to lysosomal proteolysis and quenching in acidic pH, whereas RFP is not (Sun et al., 2012). Our results showed that the expression of SQSTM1 was indeed decreased in a dose-dependent manner (Figures 2C,D). We also observed fewer green puncta than red puncta by fluorescence microscopy, indicating that AFB1-induced autophagy included the process of acidification. Additionally, CQ treatment significantly increased the level of LC3-II induced by AFB1. These results confirmed that AFB1 induced a complete autophagic process and suggested that autophagy plays a role in the disposal of intracellular AFB1. However, the location of AFB1 in autophagic organelles awaits further study.

Our analysis demonstrated that the AFB1-induced autophagic response was mediated by Beclin-1 and was also dependent on MEK/ERK. Using the MEK inhibitor PD98059, we observed that the activation of MEK/ERK in response to autophagy stimuli induced by AFB1 treatment upregulated Beclin-1. This result was consistent with a previous report that MEK/ERK regulates autophagy via regulating Beclin-1 (Wang et al., 2009).

Previous studies mainly focused on the ETs produced by neutrophils and occasionally, eosinophils, or mast cells (Aulik et al., 2012). Releasing nuclear DNA to form ETs has not been reported for MΦ until recently (Chow et al., 2010). Our team has shown that peritoneal MΦ (RAW264.7) release METs after treatment with PMA (Liu et al., 2014). In this study, we found that not only RAW264.7 cells but also THP-1 cells produced METs in response to AFB1. In particular, our results also indicated that MET formation significantly reduced the AFB1 content, whereas treatment with the METs scavenger DPI (50 μM) reversed the degradation. The results therefore revealed that phagocytes could not only recognize and treat intracellular toxic AFB1 by autophagy but also address extracellular toxic AFB1 through MET formation.

Two types of mechanisms are involved in the formation of ETs: NOX2-dependent and NOX2-independent. Our results showed that the AFB1-induced METs were NOX2-dependent, similar to those observed for PMA, because the NOX2 inhibitor DPI inhibited the formation of METs induced by AFB1. Additional assays indicated that AFB1 treatment triggered a significant increase in cytosolic ROS production in THP-1 cells (Figure 5C). Recent studies have shown that NOX2-mediated ROS production is related to the activation of autophagy by microorganisms in phagocytes (Huang et al., 2009). It has been suggested that the autophagic machinery is activated during ROS production to counter possible oxidative burst-dependent cell damage and death (Mitroulis et al., 2010).

The different environmental signals from different tissues, such as injury, healing, and repair, underlie the heterogeneity of macrophages (Ben-Mordechai et al., 2015). M1 and M2 are the most common phenotypes of macrophages. M1 macrophages are pro-inflammatory, whereas M2 macrophages are anti-inflammatory and reparative. Both macrophage types play irreplaceable roles in the immune system. Thus, the polarization of macrophages has been studied widely. In many recent reports, granulocyte-macrophage colony stimulating factor (GM-CSF), lipopolysaccharides (LPS) and interferon (IFN) γ were shown to stimulate macrophages to the M1 phenotype (Brown et al., 2012). In addition, the M2 macrophage phenotype was stimulated by macrophage colony-stimulating factor (M-CSF), IL-4, IL-10, IL-13, and glucocorticoid hormones (Biswas et al., 2012). AFB1 has high toxicity and can cause hepatic disease. Our results showed that AFB1 induced polarization of RAW264.7 cells to the M1 phenotype in a dose-dependent manner (Figure 8A and Supplementary Figure S2). Because M1 macrophages are pro-inflammatory, we speculate that AFB1 leads to liver damage by inducing M1 macrophage polarization.

Overall, this study demonstrated that MΦ could recognize or treat a certain concentration range of the exogenous toxin AFB1 with the aid of two novel innate immune responses, autophagy and ET formation. This study lays a foundation for deeper research into the innate immune mechanisms and immune control of AFB1.

## Author contributions

YA, data acquisition, data analysis, data interpretation, revising of the manuscript; XS, data acquisition, data analysis, data interpretation, revising of the manuscript; XT, data acquisition, data analysis, data interpretation; YW, data acquisition, data analysis, data interpretation; FS, data acquisition, data analysis, data interpretation; QZ, data acquisition, data analysis, data interpretation; CW, data acquisition, data analysis, data interpretation; MJ, data acquisition, data analysis, data interpretation; ML, data acquisition, data analysis, data interpretation; LY, data acquisition, data analysis, data interpretation, writing of the manuscript, revising of the manuscript, principle investigator.

### Conflict of interest statement

The authors declare that the research was conducted in the absence of any commercial or financial relationships that could be construed as a potential conflict of interest.

## Supplementary material

Figure S1 **METs was stained with SYTOX Orange (5 mM) for 10 min and Hoechst 33342 (1 μM) for 5 min**. The images were obtained by fluorescence microscopy with a 20 × objective lens. Scale bars = 50 μm.

Figure S2 **(A)** CCR7 and CD163, CD86 and CD206 with eDNA [stained with Hoechst 33342 (1 μM) for 5 min] colocate in RAW264.7 cells. The images were obtained by fluorescence microscopy with a 20 × objective lens. Scale bars = 50 μm. **(B)** CCR7 and CD163 in RAW264.7 cells, were detected by flow cytometry. The results are presented as mean fluorescence intensity (MFI). ^***^*P* < 0.001 compared with the control groups in RAW264.7 cells. **(C)** CCR7 and CD163 in RAW264.7 cells, Atg7-silenced cells, and cells transfected with the shRNA negative control were detected by flow cytometry. The results are presented as the mean fluorescence intensity (MFI). ^***^*P* < 0.001 compared with the control groups of RAW264.7 cells; ^###^*P* < 0.001 compared with AFB1-infected cells and cells transfected with the negative control shRNA plasmid. The histogram figure represents the comparison of the 2 μM AFB1-treated group, Atg7-silenced cells, cells transfected with the shRNA negative control and control group.
