# Supplementary figures and images for: Aflatoxin B1 Induces Reactive Oxygen Species-Mediated Autophagy and Extracellular Trap Formation in Macrophages
Source: Front Cell Infect Microbiol. 2017 Feb 23;7:53. doi: 10.3389/fcimb.2017.00053 (PMC5322174; doi:10.3389/fcimb.2017.00053)

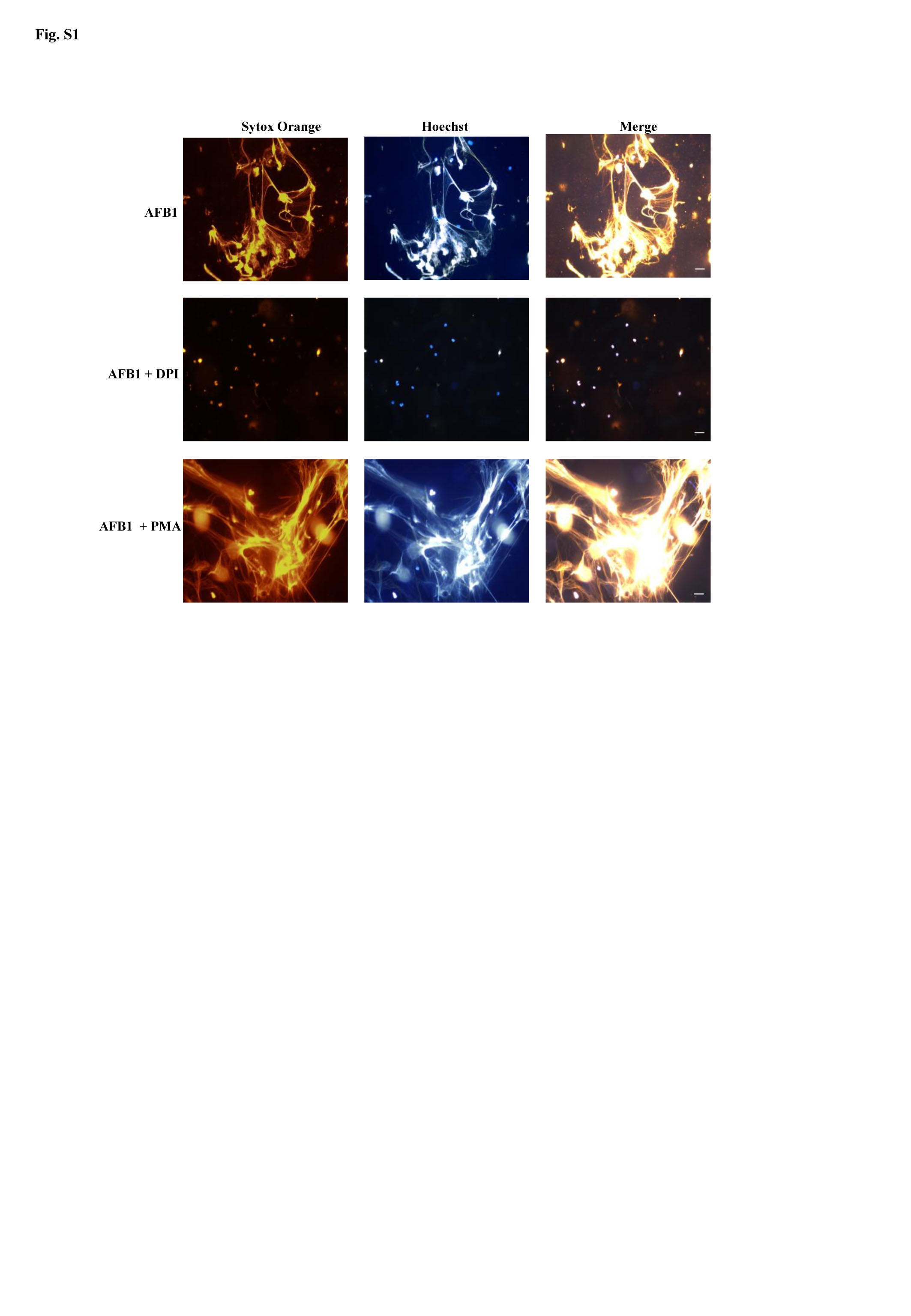

Supplement: Figure S1 — METs was stained with SYTOX Orange (5 mM) for 10 min and Hoechst 33342 (1 μM) for 5 min. The images were obtained by fluorescence microscopy with a 20 × objective lens. Scale bars = 50 μm. [file Image1.JPEG]

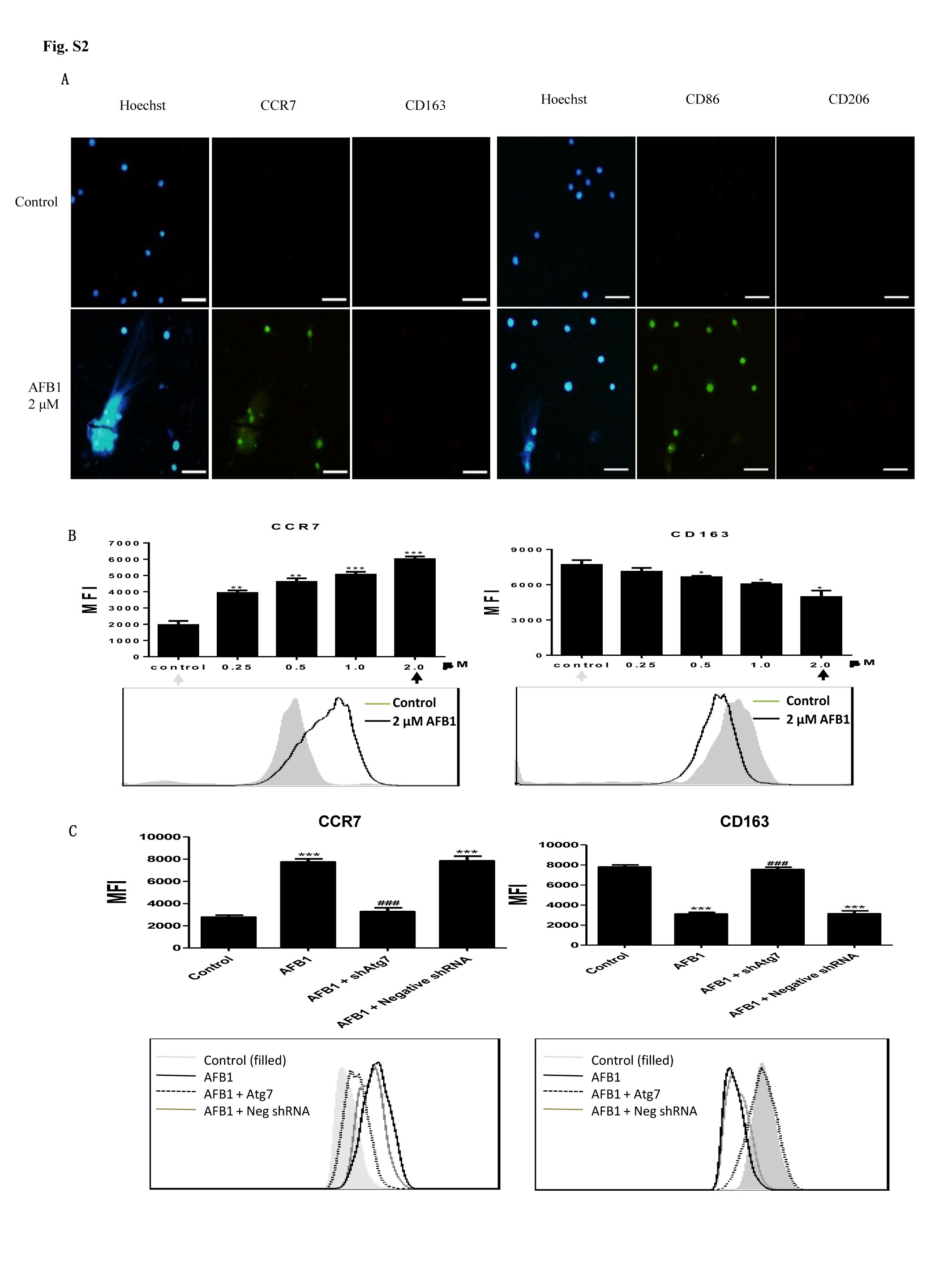

Supplement: Figure S2 — (A) CCR7 and CD163, CD86 and CD206 with eDNA [stained with Hoechst 33342 (1 μM) for 5 min] colocate in RAW264.7 cells. The images were obtained by fluorescence microscopy with a 20 × objective lens. Scale bars = 50 μm. (B) CCR7 and CD163 in RAW264.7 cells, were detected by flow cytometry. The results are presented as mean fluorescence intensity (MFI). ***P < 0.001 compared with the control groups in RAW264.7 cells. (C) CCR7 and CD163 in RAW264.7 cells, Atg7-silenced cells, and cells transfected with the shRNA negative control were detected by flow cytometry. The results are presented as the mean fluorescence intensity (MFI). ***P < 0.001 compared with the control groups of RAW264.7 cells; ###P < 0.001 compared with AFB1-infected cells and cells transfected with the negative control shRNA plasmid. The histogram figure represents the comparison of the 2 μM AFB1-treated group, Atg7-silenced cells, cells transfected with the shRNA negative control and control group. [file Image2.JPEG]
